# A Weighted Survival Regression Framework for Incorporating External Prediction Information

**DOI:** 10.1007/s42519-025-00471-1

**Published:** 2025-07-25

**Authors:** Debashis Ghosh

**Affiliations:** Department of Biostatistics and Informatics, Colorado School of Public Health, Aurora, CO 80045 USA

**Keywords:** Additive hazards, Proportional hazards, Risk prediction, Semiparametric regression, Survival analysis

## Abstract

In this article, we develop a weighted approach to estimation for right-censored time to event data in the presence of external predictions available from a prediction model. There are several advantages to the proposed approach. First, the method allows for arbitrary forms for the external prediction model. Second, the methodology can be fit easily using standard software packages that allow for subject-specific weights. Third, all that is needed from the external models are access to predictions and not the actually prediction equation. A complication is that inference becomes challenging, so we develop new theoretical results along with a perturbation-based method for inference. The methodology is applied to three publicly available datasets.

## Introduction

There has been tremendous interest in the use of incorporating external data sources in analyses of local datasets. We use the latter term to refer to the dataset that the analyst is working with at hand. In an ideal scenario, the individual-level data from all sources are available so that meta-analytic approaches can be performed. For many clinical situations, individual-level external data are not available; this could be due to logistical, ethical or practical constraints. Thus, the external information can come in the form of summary statistics, parameter estimates or prediction equations. How to perform modeling in this scenario has been of recent interest in the statistical literature. Chatterjee et al. [3] proposed the use of constrained maximum likelihood methods in which the parameters from the external data source served as parameter constraints on the likelihood for the local dataset. The discrepancies between the constraints and model could be resolved by appealing to an argument based on minimizing Kullback-Leibler divergence. However, a key factor to the success of this approach is whether or not the covariate distribution used to build the calculator is the same as that in the local dataset. If this assumption is violated, Chatterjee et al. [3] proposed the use of a synthetic constrained maximum likelihood to account for differences in covariate distributions. More recently, Taylor, Mukherjee and colleagues have proposed a variety of approaches to this problem [5, 6, 13] that exploit constrained estimation, Bayesian and empirical Bayesian methods.

For the survival setting, there has been important work by Qin, Huang and collaborators on the topic [4, 19, 20]. Using empirical likelihood, these authors considered estimation of semiparametric survival models in which the external information was incorporated as constraints. Huang et al. [20] proposed a double empirical likelihood method in which constraints defined by the proportional hazards model and the external information were placed. This was extended in Chen et al. [4] to allow for incompatibility of information through an extra set of constraints that measured the compatibility that had an associated set of parameters. This could then be jointly estimated using empirical likelihood in a coherent manner. The theory of empirical likelihood is quite general and allows for a pretty general estimation framework. Under certain regularity conditions, the resulting parameter estimates are consistent and asymptotic normal. He et al. [18] adapted the methodology in Huang et al. [20] to develop an approach to incorporating parameter estimation for the additive hazards model, discussed in §2.4. Related to empirical likelihood is the generalized method of moments procedure, and Huang and Qin [19] use this approach for estimating the proportional hazards regression model while incorporating the external information.

In this article, we develop a general framework for survival regression estimation in the presence of external information. There are several advantages to the proposed framework:

1. The framework can accommodate both proportional hazards and additive hazards regression models.
2. The approach can be fit using most standard software packages that allow for weights.
3. The procedure allows for the variables in the external and local calculators to be different.
4. The conditions for validity of our procedure are far less than those proposed in the previous paragraphs. In particular, it does not require fitting the true model to the data.

While these are very appealing features of the methodology, there are two complications that arise from using our proposed approach. First, inference is not straightforward. This will necessitate new developments based on the theory of model misspecification for survival regression models [26, 38] in conjunction with the perturbation approach of Jin et al. [22]. Second, because we are fitting known misspecified models to the data, we can only describe our results in terms of measures of associations and not relative risk estimates (as in proportional hazards models) or risk differences (as in additive hazards models). We note that a related methodology appears in Ghosh and Sabel [15], but that procedure is not directly adapted to the survival data problem, nor was there theoretical justification presented. The structure of the paper here is as follows. In Section 2, we provide a high-level description of the methodology along with the application and implementation to the proportional hazards and additive hazards regression models. Several numerical examples are presented in Section 3. Finally, some discussion concludes Section 4.

## Proposed Methodology

### High-level Description

For $i=1,\ldots ,n$, we let $\hat{w}_i(t)$ denote the estimated weight function for subject *i* at time t. This weight function comes from the external prediction model. We next define $\hat{M}_i(t;\beta )$ to denote the estimated martingale process used to fit working survival regression models to the observed data. Our approach consists of solving the equations:

1 $$\begin{aligned} \sum _{i=1}^n \int _0^{\infty } \hat{w}_i(t) dM_i(t;\beta )= &   0. \nonumber \\ \sum _{i=1}^n \int _0^{\infty } \hat{w}_i(t) Z_i dM_i(t;\beta )= &   0, \end{aligned}$$

where $Z_i$ $(i=1,\ldots ,n)$ generically denotes the covariates. It is well-known that for both the proportional and additive hazards models, the estimation of regression coefficients can be framed using (1), which will be described in subsequent sections. In each of the following subsections, we will show that the left-hand side of (1), normalized by its expectation, converges uniformly to its expectation under the true data-generating distribution. If we let the solution to (1) be denoted as $\hat{\beta }$, then $\hat{\beta }$ will converge to the least false parameter $\beta ^*$ in the sense of Struthers and Kalbfleisch [38].

Next, we generate iid random variables $G_i$ $(i=1,\ldots ,n)$ with mean *g* and variance one. Define the perturbed equations

2 $$\begin{aligned} \sum _{i=1}^n \int _0^{\infty } \hat{w}_i(t) (G_i - g) dM_i(t;\beta )= &   0. \nonumber \\ \sum _{i=1}^n \int _0^{\infty } \hat{w}_i(t) Z_i (G_i - g) dM_i(t;\beta )= &   0. \end{aligned}$$

Define the solution to (2) by $\tilde{\beta }$. Then, by using the arguments in Parzen et al. [34], we have that the conditional distribution of $n^{1/2}(\tilde{\beta }- \hat{\beta })$, given the data approximates the unconditional distribution of $n^{1/2}(\hat{\beta }- \beta ^*)$. This yields the following perturbation algorithm for constructing confidence intervals for $\beta ^*$:

1. Solve (1) to obtain $\hat{\beta }$.
2. Generate iid random variables $G_i$ $(i=1,\ldots ,n)$ with mean *g* and variance one.
3. Using $$G_1,\ldots ,G_n$$, solve (2).
4. Repeat steps 2. and 3. *B* times.
5. Use the *B* solutions of (2) to construct the empirical distribution for $\tilde{\beta }$.

For the steps in the algorithm, step (1) yields the estimate of the regression coefficients based on the observed data. Next, steps (2) – (4) represents a resampling step akin to using the nonparametric bootstrap [12]. However, the perturbation scheme we describe is easily amenable to fitting in standard software packages that allow for subject-specific weights. Based on the empirical distribution of $\tilde{\beta }$, we can use the percentile method for constructing confidence intervals. Given a confidence level $(1-\alpha )$, we use the $\alpha /2$th and $100(1-\alpha /2)$th percentiles of the empirical distribution for $\tilde{\beta }$ to obtain pointwise confidence limits. This would correspond to a bootstrap percentile interval as described in Efron and Tibshirani [12]. Note that *B* plays a role akin to the number of bootstrap samples for the nonparametric bootstrap. In practice, we can take $B = 500$ or $B = 1000$.

Before delving into the approaches for the proportional and additive hazards models, we make a few remarks. First, the information from external risk prediction models is manifested through the weights. We only need access to a risk prediction model that provides predictions. The model could be parametric or based on machine learning. Second, this approach avoids model compatibility issues that are implicit in previous parameter sharing approaches. Third, the asymptotic results are predicated on a theory of model misspecification, so there is minimal reliance on the risk prediction or the model specified correct or true. This mimics the ‘working model’ ideas in the work of Cai, Tian and colleagues [2, 41]. Fourth, the approach is very easy to implement and can be done using standard software packages that allows for weighted observations for individuals. Note that to implement this in software, we use the optimization-based approach to perturbation described in Jin et al. [22]. We present examples in §3 to illustrate the methodology and provide attendant R code for the example in §3.3, available at github.com/GhoshLab/WeightedSurv, for dissemination purposes.

### Role of Weight Function

A natural question that arises is how to choose the weight function in (2). We think the choice needs to be guided by the goal of the analysis and in particular, what observations should be given relatively more weight in the analysis. For the current paper, we give larger weights to observations with higher risk of the event.

An alternative goal might be to perform incremental value analyses of additional biomarkers relative to existing models [17, 35]. For those situations, we would want to give more weight to observations that have poor survival. In our experience, when using this approach, we have found that the standard errors become higher and that inference is more conservative. Intuitively, this is reasonable, as the poorly predicted observations are given higher weight; these are the subjects which are discordant with the prediction model. This is analogous to a Bayesian framework in which information from the likelihood and prior conflict, which leads to inconclusive evidence.

### Application to Proportional Hazards Model

We first assume that the analyst wishes to fit the proportional hazards regression model to the data. Define *T* to be the event time and *C* to be the censoring time. The observed followup time is $X = \min (T,C)$, and the event indicator is $\delta = I(T \le C)$. We let *Z* denote a $p-$dimensional vector of covariates. For $i=1,\ldots ,n$, the observed data consist of $(X_i,\delta _i,Z_i)$, iid observations from $(X,\delta ,Z)$. If we define $\lambda (t|Z)$ to be the conditional hazard function at time *t* given covariates, the proportional hazards model is given by

3 $$\begin{aligned} \lambda (t|Z) = \lambda _0(t)\exp (\beta 'Z), \end{aligned}$$

where $\lambda _0(t)$ is a baseline hazard function and $\beta$ represents a $p-$dimensional vector of regression coefficients. As is well-known in the literature, the partial likelihood [9] can be maximized to find an estimator $\hat{\beta }$ for $\beta$ in (3). Given $\hat{\beta }$, an estimate of the cumulative baseline hazard function can be computed using the Aalen-Breslow estimator.

For $i=1,\ldots ,n$, define the counting processes $N_i(t) = I(X_i \le t, \delta _i = 1)$. Applying the equations (1), we can express the partial likelihood solution to the Cox model as the solution to the following estimating equation: $U_c(\beta ) = 0,$ where

$$ U_c(\beta ) = \sum _{i=1}^n \int _0^{\infty } \hat{w}_i(u) \left\{ Z_i - \frac{S^{(1)}(\beta ,u)}{S^{(0)}(\beta ,u)} \right\} dN_i(u),$$

where

$$ S^{(k)}(\beta ,t) \equiv \sum _{i=1}^n I(X_i \ge t)Z_i^{(k)}\exp (\beta 'Z_i),$$

$a^{(1)} = a$ and $a^{(0)} = 1$. Large-sample properties of $\hat{\beta }$ have been elegantly established using martingale theory, as found in Fleming and Harrington [14].

Note the similarity of $U_c(\beta )$ to (1). Our proposed approach to incorporating external risk prediction information for the proportional hazards model proceeds as follows:

1. Using the external risk prediction, obtain weights for the *i*th subject, $\hat{w}_i$, $i=1,\ldots ,n$;
2. Fit a proportional hazards model to the dataset $(X_i,\delta _i,Z_i)$, $i=1,\ldots ,n$ with weights $\hat{w}_i$; this can be done in R using the survival package [40];
3. Generate a random sample of *n* exponential random variables $V_1,\ldots ,V_n$;
4. Fit a proportional hazards model to the dataset $(X_i,\delta _i,Z_i)$, $i=1,\ldots ,n$ with weights $G_i \equiv V_i\hat{w}_i$;
5. Repeat steps 3. and 4. B times;
6. Using the empirical distribution of the solutions of the *B* proportional hazard estimates, compute confidence intervals using the percentile method.

We note that because the $G_i$ $(i=1,\ldots ,n)$ have mean one, the unconditional distribution of $\hat{\beta }$, suitably centered and normalized, can be approximated by the conditional distribution of $\tilde{\beta }$ , suitably centered and normalized. We are conditioning on the observed data, and thus the distribution of $\tilde{\beta }$ resembles those generated using the bootstrap [12].

Next, we prove theoretical results for the proposed methodology with the proportional hazards model. For $i=1,\ldots ,n$, define the counting process $N_i(t) = I(X_i \le t, \delta _i = 1)$ and the at-risk process $Y_i(t) = I(X_i \ge t)$. We assume the following regularity conditions:

1. $\{N_i(\cdot ),Y_i(\cdot ),Z_i\}$ are independent and identically distributed observations;
2. There exists a constant $\tau > 0$ such that $P(C_i> \tau ) >0$ for $i=1,\ldots ,n$.
3. The covariates $Z_i$ $(i=1,\ldots ,n)$ are bounded.
4. The weight functions $\hat{w}_i(t)$ converge uniformly in *t* to deterministic functions $w_i(t)$ uniformly in $t \in [0,\tau ]$ for $i=1,\ldots ,n$.

#### Theorem 1

Assuming conditions [R1]–[R4], the solution to $U_c(\beta ) = 0$, $\hat{\beta }$, converges in probability to $\beta ^*$. The definition of $\beta ^*$ is defined in the theorem.

#### Proof

Define

$$ S^{(r)}(\beta ,t) = n^{-1} \sum _{i=1}^n Y_i(t)Z_i^{\otimes r}\exp (\beta 'Z_i)\hat{w}_i(t),$$

where $a^{\otimes 0} = 1$, $a^{\otimes 1} = a$, and $a^{\otimes 2} = aa'$, and $s^{(r)}(\beta ,t) = E\{S^{(r)}(\beta ,t)\}$. In addition, we define $\lambda _i(t)$ to represent the true hazard function for subject *i*, $i=1,\ldots ,n$. In addition, we define

$$ S^{(r)}(t) = n^{-1} \sum _{i=1}^n Y_i(t)Z_i^{\otimes r}\lambda _i(t)\hat{w}_i(t),$$

and $s^{(r)}(t) = E\{S^{(r)}(t)\}$, $r=0,1,2$. Then by applying repeated applications of the functional law of large numbers [36] in conjunction with the continuous mapping theorem, $n^{-1} U_c(\beta )$ converges almost surely to $u(\beta )$, where

$$ u(\beta ) = \int _0^{\tau }s^{(1)}(t) - \int _0^{\tau }\frac{s^{(1)}(\beta ,t)}{s^{(0)}(\beta ,t)} s^{(0)}(t).$$

Adapting the proofs of Lemma 3.1. and Theorem 4.2. from  Andersen and Gill [1], we have that under regularity conditions [R1.]–[R4.], $\hat{\beta }$ will converge almost surely to $\beta ^*$, the unique solution of $u(\beta ) = 0$. This also requires $A(\beta )$ to be positive definite, where

$$ A(\beta ) = \int _0^{\tau }\left\{ \frac{s^{(2)}(\beta ,t)}{s^{(0)}(\beta , t)} - \frac{s^{(1)}(\beta ,t)^{\otimes 2}}{s^{(0)}(\beta , t)^2} \right\} s^{(0)}(t) dt.$$

$$\square $$

#### Theorem 2

The limiting distribution of $n^{1/2}(\hat{\beta }- \beta ^*)$ is a $p-$dimensional normal random vector with mean zero and variance covariance matrix

$$ A(\beta )^{-1}B(\beta )A(\beta )^{-1},$$

where

$$ B(\beta ) = \lim _{n \rightarrow \infty } n^{-1} \sum _{i=1}^n V_i(\beta ),$$

where for $$i=1,\ldots ,n$$,

$$\begin{aligned} V_i(\beta )= &   \hat{w}_i(X_i)\delta _i \left\{ Z_i - \frac{S^{(1)}(\beta ,X_i)}{S^{(0)}{(\beta ,X_i)}} \right\} - \sum _{j=1}^n \frac{\delta _j\hat{w}_j(X_j)Y_j(X_i)\exp (\beta 'Z_i)}{nS^{(0)}(\beta ,X_j)} \\    &   \times \left\{ Z_i - \frac{S^{(1)}(\beta ,X_j)}{S^{(0)}(\beta ,X_j)} \right\} . \end{aligned}$$

#### Proof

Performing a Taylor series expansion of $U_c(\hat{\beta }) = 0$ around $\beta ^*$, we have

$$\begin{aligned} 0= &   U_c(\hat{\beta }) \\= &   U_c(\beta ^*) + (\hat{\beta }- \beta ^*)\frac{\partial }{\partial \beta }U_c(\beta ^*) \\  &   + R_n, \end{aligned}$$

where $R_n$ is a remainder term that under regularity conditions (R1.)-(R4.) converges in probability to zero at an $n^{1/2}$ rate. By the central limit theorem and (R1.)-(R4.), $n^{-1/2}U_c(\beta ^*)$ converges to a zero-mean normal distribution with covariance matrix $B(\beta )$. Since $n^{-1} \partial U_c/\partial \beta$ converges in probability to $A(\beta )$ by the weak law of large numbers, combining these results plus application of Slutsky’s theorem yields that $n^{1/2}(\hat{\beta }- \beta ^*)$ converges in distribution to a normal distribution with variance $A(\beta )^{-1}B(\beta )A^{-1}(\beta )$. In principle, we could estimate the variance of $n^{1/2}(\hat{\beta }- \beta ^*)$ consistently using

$$A(\beta ^*)^{-1}B(\beta ^*)A^{-1}(\beta ^*).$$

We instead use the perturbation algorithm described earlier. $\square$

### Application to Additive Hazards Model

An alternative to proportional hazards regression is the so-called additive hazards model [27, 31]:

4 $$\begin{aligned} \lambda (t|Z) = \lambda _0(t) + \alpha 'Z, \end{aligned}$$

where $\alpha$ is a $p-$dimensional vector of unknown regression coefficients. As argued by Lin and Ying [27], there are certain advantages of the additive hazard model: (1) interpretation of $\alpha$ as a risk difference, which has important implications for public health and causal inferential settings; (2) conceptual links to linear regression, which is perhaps the canonical regression modelling procedure in statistics.

For the additive hazards model, with $i=1,\ldots ,n$,

5 $$\begin{aligned} M_i(t;\alpha ) \equiv N_i(t) - I(X_i \ge t)\{\lambda _0(t) + \alpha 'Z\}dt \end{aligned}$$

will be a zero-mean martingale at the true values of $\alpha$ and $\lambda _0(t)$ under correct model specification. Lin and Ying [27] apply the equations in (1) to derive a closed-form estimator of $\hat{\alpha }$ and derive its consistency and asymptotic normality using martingale theory.

For our problem, we use the following algorithm, which is very similar to the one in the previous section:

1. Using the external risk prediction, obtain weights for the *i*th subject, $\hat{w}_i$, $i=1,\ldots ,n$
2. Fit an additive hazards model to the dataset $(X_i,\delta _i,Z_i)$, $i=1,\ldots ,n$ with weights $\hat{w}_i$; this can be done in R using the timereg package [31].
3. Generate a random sample of *n* exponential random variables $V_1,\ldots ,V_n$;
4. Fit an additive hazards model to the dataset $(X_i,\delta _i,Z_i)$, $i=1,\ldots ,n$ with weights $G_i \equiv V_i\hat{w}_i$;
5. Repeat steps 3. and 4. B times;
6. Using the empirical distribution of the solutions of the *B* proportional hazard estimates, compute confidence intervals using the percentile method.

This algorithm mimics what was done in the proportional hazards regression case in §2.3. However, we note that the numerical algorithm used to solve for $\alpha$ here differs from the partial likelihood optimization used for the proportional hazards model. Next, we will prove theoretical results about our proposed estimation procedure analogous to Theorems 1 and 2:

#### Theorem 3

Assuming regularity conditions [R1.]–[R4.], the estimator $\hat{\alpha }$ converges to $\alpha ^*$, the solution to $\tilde{u}(\alpha ) = 0$, where

$$ \tilde{u}(\alpha ) = E\left[ \int _0^{\tau }(Z_1 - \bar{z}(t))dM^*(t;\alpha )\right] = 0,$$

$$\bar{z}(t) = \lim _{n \rightarrow \infty } \frac{\sum _{j=1}^n Y_j(t)Z_j}{\sum _{j=1}^n Y_j(t)},$$

and $M^*(t;\alpha ) = N(t) - \Lambda _*(t;\alpha )$, where $\Lambda _*$ denotes the cumulative hazard function of the true model. Note that the expectation in the definition of $\tilde{u}(\alpha )$ is taken with respect to the true model.

#### Proof

From Lin and Ying [27], we observe that $\hat{\alpha }$ is the solution to $U_1(\alpha ) = 0$, where

$$ U_1(\alpha ) = n^{-1} \sum _{i=1}^n \int _0^{\tau }(Z_i - \bar{Z}(t)) \{dN_i(t) - Y_i(t)\alpha 'Z_i dt\},$$

where

$$ \bar{Z}(t) = \frac{\sum _{j=1}^n Y_j(t)Z_j}{\sum _{j=1}^n Y_j(t)}.$$

Application of the functional strong law of large numbers [36] shows that $U_1(\alpha )$ converges to $\tilde{u}(\alpha )$. By regularity conditions [R1.]–[R4.], we have that the solution to $\tilde{u}(\alpha ) = 0$, $\alpha ^*$, will be unique provided that

$$ C \equiv E \left\{ \int _0^{\tau }[Z_1 - \bar{z}(t)]^{\otimes 2}dN_i(t)]\right\} $$

is positive definite. This will be the case if the components of *Z* are linearly independent.

Adapting the proof of Theorem 2, it is straightforward to demonstrate the asymptotic normality of $n^{1/2}(\hat{\alpha }- \alpha ^*)$. $\square$

#### Theorem 4

Assuming regularity conditions [R1.]–[R4.] hold, $n^{1/2}(\hat{\alpha }- \alpha ^*)$ converges in distribution to a mean zero normal random vector with variance covariance matrix $D^{-1}ED^{-1}$, where

$$\begin{aligned} D= &   \lim _{n \rightarrow \infty } n^{-1} \sum _{j=1}^n \frac{\partial }{\partial \alpha } U_{1j}(\alpha ^*)\\ E= &   E[\tilde{u}(\alpha ^*)\tilde{u}(\alpha ^*)']. \end{aligned}$$

As in the proportional hazards case, the matrices *D* and *E* can be consistently estimated using the observed data.

### Heuristics for Perturbation Scheme

For both the proportional hazards and additive hazards regression models, we have proven consistency and asymptotic normality results in Theorems 1 – 4. As described in the algorithms for both procedures, we will generate exponential weights that function as bootstrap-like perturbations, with which we repeatedly solve for the regression coefficient estimators.

Our justification comes from the work of Jin et al. [22]. For both procedures, we are optimizing with respect to an objective function. It is the partial likelihood for the proportional hazards model and least squares for the additive hazards model. Denote the objective function contribution for the *i*th subject by $L_i$ for both models. We use $\theta$ to generically denote the regression coefficient from the previous section ($\beta$ for proportional hazards, $\alpha$ for the additive hazards model). For both estimators, we have demonstrated that

$$ n^{1/2}(\hat{\theta }- \theta ^*) \rightarrow _d N(\textbf{0},\textbf{M}),$$

where the form of $\textbf{M}$ is given in Theorems 2 and 4.

From Jin et al. [22], the Taylor series expansions used for the proofs of Theorems 2 and 4 implies that their assumption (A.1) holds in the current, paper. We have

6 $$\begin{aligned} \sqrt{n}(\hat{\theta }- \theta ^*) = -DW_n(\theta ^*) + o(1 + \Vert W_n(\theta )\Vert + n^{1/2}\Vert \hat{\theta }- \theta ^*\Vert ), \end{aligned}$$

where $-D$ is the inverse of the Fisher information matrix and

$$W_n(\theta _0) \equiv n^{-1/2}\sum _{i=1} (\partial /\partial \theta ) log L_i(\theta )$$

is the normalized score function. Regularity conditions [R1.]–[R4.] and Theorems 1 and 3 guarantee that the remainder term on the right-hand side of (6) converges to zero in probability. Thus Proposition (A2) from Jin et al. [22] holds as well. We can repeat the same arguments using $\tilde{l}(\theta ),$ where

$$\tilde{l}(\theta ) = \sum _{i=1}^n G_i \frac{\partial }{\partial \theta } log L_i(\theta ),$$

where we condition on $G_1,\ldots ,G_n$. This will yield that conditional on the observed data and the $G'$s,

7 $$\begin{aligned} \sqrt{n}(\tilde{\theta }- \hat{\theta }) = -D \hat{W}_n(\hat{\theta }) + o(1 + \Vert W_n(\hat{\theta })\Vert + n^{1/2}\Vert \tilde{\theta }- \hat{\theta }\Vert ), \end{aligned}$$

where

$$\hat{W}_n(\theta ) = n^{-1/2}\sum _{i=1}^n w_i (\partial /\partial \theta ) log L_i(\hat{\theta }).$$

The equivalences of (6) (unconditionally) and (7) (conditional on the data) means that the unconditional distribution of $n^{1/2}(\hat{\theta }- \theta ^*)$ and the conditional distribution $n^{1/2}(\tilde{\theta }- \hat{\theta })$ will asymptotically converge to the same limit. This is in line with the theory of the bootstrap [12] and justifies our proposed resampling schemes.

## Numerical Examples

Throughout the examples, we will use two approaches to building risk prediction models for external information: proportional hazards regression and random survival forests [21]. For the weight from the proportional hazards models, we will use the predicted survival probability at 5 years. All examples used $B = 1000$.

### Primary Biliary Cirrhosis Data

**Fig. 1:**
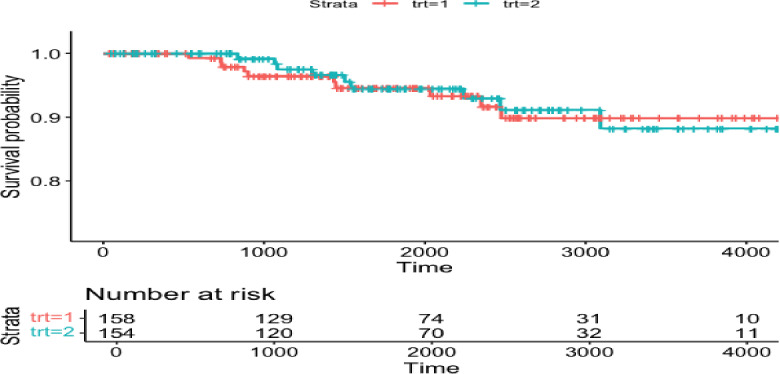
Survival plot in PBC data. Trt = 1 denotes penicillamine, and Trt = 2 denotes placebo

Our first example is the very famous primary biliary cirrhosis (PBC) dataset from Fleming and Harrington [14]. The main dataset that has been considered in the literature represent the observations that are from the randomized clinical trial reported in Dickson et al. [11]. To contextualize the study, a major question in the 1970s was how to treat PBC patients, which represents an end-stage disease. In particular, the main treatment option available to these patients is liver transplantation. Given the scarcity of available liver donors, an important question becomes when to perform liver transplantation in this population [16]. To do this required understanding the natural history of the disease. From 1974 to 1984, Dickson et al. [11] performed a clinical trial in 312 subjects were randomized to two arms: (1) the treatment arm, consisting of 1 g/penicillamine on a daily basis; (2) the control arm, which was placebo. However, 106 patients declined randomization and thus represent an observational arm. Most papers in the statistical literature use data on the 312 patients who were randomized. Here, we use the 106 patients in the observational arm to build a natural history model of risk prediction for survival, and then apply the weighted procedure to the data in the randomized clinical trial using weights based on the risk prediction model. The main goal here will be to evaluate the treatment effect, which was reported to have a slightly beneficial but statistically insignificant effect in the original clinical trial [11]. The plot of the survival curves by treatment in the RCT is shown in Figure  1.

Our first set of analyses uses the proportional hazards model to estimate the treatment effect in the clinical trial dataset. We build risk prediction models for the weights using the 106 patients in the observational arm in two ways: (a) using a proportional hazards model; (b) using survival random forests [21]. To implement the approach, we need use variables available in both the RCT and the observational groups. We use the following variables to build the risk prediction models: sex, age, edema, bilirubin and albumin.

As a baseline analysis, we estimated the treatment effect in the RCT data only using no external information using the proportional hazards model. Doing this yielded a log hazard ratio of $-0.064$ with an attendant standard error of 0.460. Next we used the external model built using the Cox proportional hazards model and used the weights based on predicted survival. This yielded a log hazard ratio of $-0.035$ with an attendant standard error of 0.454. Finally, we used survival random forests for prediction [21], where the cumulative hazard function was used to construct weights. For this approach, we obtained a log hazard ratio of $-0.134$ with an attendant standard error of 0.454. We repeated the same analyses using the additive hazards model as the model to fit to the data; the results are shown in Table 1. While none of the results are significant, we see that incorporating the weights leads to larger effect sizes relative to the no weights approach.

**Table 1:** Additive hazards results for the PBC data

Method	Est. Coef	SE	p-value
No weights	$-1.09\times 10^{-6}$	$1.38\times 10^{-5}$	0.89
PH weights	$-1.08\times 10^{-6}$	$1.38\times 10^{-5}$	0.94
RF weights	$-6.29\times 10^{-6}$	$1.94\times 10^{-5}$	0.74

### Radiomics in Head and Neck Cancer Example

Our second example seeks to evaluate the utility of a radiomics-based predictor for survival in head and neck cancer patients. Radiomics explores the relationships between the image-derived characteristics of a tumor and other parameters, including clinical outcomes and genomic profiles, including gene expression, somatic mutations, and DNA methylation [32]. In particular, several groups have built classifiers to predict tumor molecular phenotypes using radiomic inputs (e.g., [23, 37, 44, 46]). More recently, there has been tremendous interest in the use of modern machine learning and in particular deep learning tools to build state-of-the-art classifiers for predictions [24, 25, 33]. Many of these approaches do not handle censored outcome variables, as noted in Suresh et al. [39].

In this section, we will use data from van Dijk et al. [42] to evaluate radiomics information as a predictor of survival. The data are from patients who underwent treatment for head and neck cancer at MD Anderson Cancer Center as part of their Big Data Radiotherapy HNC collection effort. The patients were included for the cohort if they satisfied the following criteria: (1) proven squamous cell carcinoma of the head and neck, (2) treatment with definitive or adjuvant radiotherapy with/without chemotherapy, and (3) no prior head and neck radiation. There are two datasets, one with imaging features, and the other without. There are 438 and 3027 observations, respectively, in the two datasets.

**Fig. 2:**
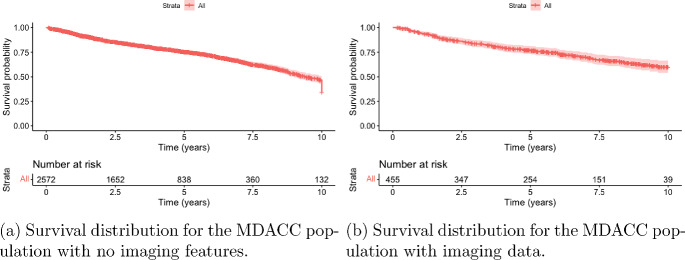
Kaplan-Meier estimators of the survival distributions of time to death for two MDACC subcohorts: (a) subjects who were not profiled by imaging; (b) subjects who were profiled by imaging

**Fig. 3:**
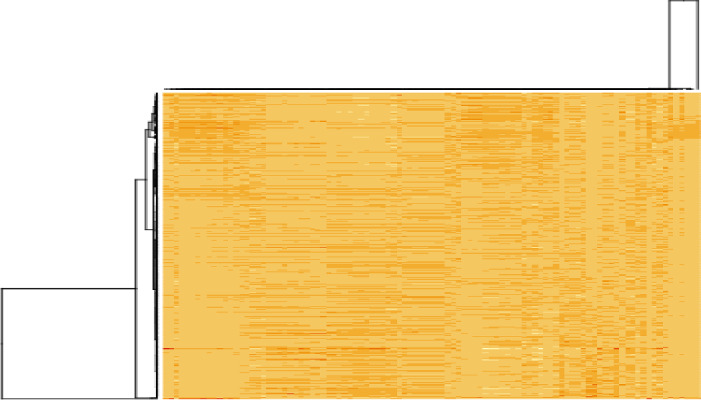
Correlation structure of the radiomics features in the MDACC dataset. The rows of the matrix represent the samples, while the columns are the radiomic features. The columns of the matrix have been scaled for the purposes of visualization. The more red the values, the stronger the correlation

We built the risk prediction model to predict survival using age, sex and T-stage. This was done using the proportional hazards regression model and random survival forests. We note that age was categorized as the following: $< 55$, $55 - 65$, $65 - 75$ and $> 75$. We will fit models on the local dataset using the following variables: age, sex, smoking status, packyears and T-stage. The correlation plot of the radiomics data across samples is shown in Figure 2. Based on the correlation structure in Figure 3, we chose to use the first principal component to summarize the information in the imaging data. We first present the results fitting proportional hazards regression to the local dataset.

**Table 2:** Proportional hazards regression results for Head and Neck Cancer Data

Variable	Unweighted		PH-weighted		RSF-weighted	
	Estimate	SE	Estimate	SE	Estimate	SE
Radiomics Score	0.23	0.15	0.05	0.02	0.24	0.12
Age (55-65)	0.21	0.23	0.47	0.32	0.11	0.28
Age (65-75)	0.55	0.25	1.23	0.33	0.62	0.27
Age > 75	1.45	0.38	2.08	0.46	1.41	0.36
Male	-0.16	0.25	0.33	0.25	0.36	0.31
T-stage 3	0.40	0.22	0.87	0.21	0.29	0.23
T-stage 4	0.85	0.23	0.99	0.35	1.03	0.27
Smoking status (never)	-0.41	0.27	-0.69	0.36	-0.53	0.34
Pack-years	0.008	0.003	-0.001	0.005	0.004	0.004

Based on the results from Table 2, we see some evidence of differences in the parameter estimates between the unweighted and weighted approaches. These are manifested as changes in effect direction (e.g., Sex) or in effect magnitude. However, the random survival forests weights appear to be closer to the unweighted analyses than the PH-weighted analyses. We next repeated the analyses with additive hazards regression; those results can be found in the Appendix. The qualitative findings from the additive hazards regression approach mirror those from the proportional hazards models in Table 2.

### A New Staging Scheme in in Liver Cancer

Our third example comes from hepatocellular carcinoma (HCC). It is the most common type of primary liver cancer in adults and currently represents the most common cause of death in cirrhosis-affected subjects [30]. While the standard staging is done using the AJCC guidelines, the Barcelona staging approach [29] has been proposed as an alternative means of staging and treating liver cancer patients. It was based on a series of randomized clinical trials and cohort studies conducted by oncologists in Europe. With Barcelona staging, the staging scheme leads to explicit treatment options. For patients in stage 0, they are candidates for early-stage resection. Stage A represents an early-stage HCC that will be treated with resection and ablation, liver transplantation, or percutaneous treatments. For HCC patients classified as stage B, they will be treated with chemotherapy. Stage C in the Barcelona staging system represents a level at which patients would be eligible for new treatments. Finally, for stage D patients, they represent end-stage patients who are treated curatively.

**Fig. 4:**
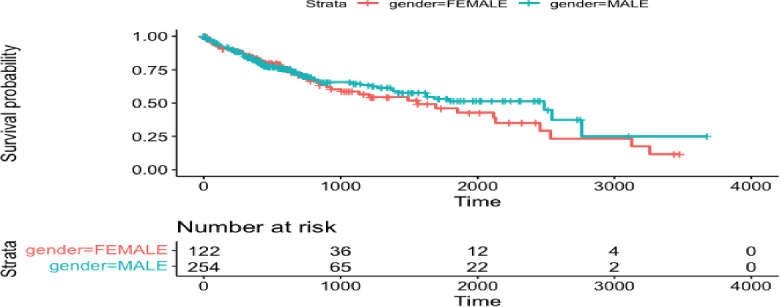
Plot of time to death by gender in TCGA liver cancer data from Liu et al. [28]

**Fig. 5:**
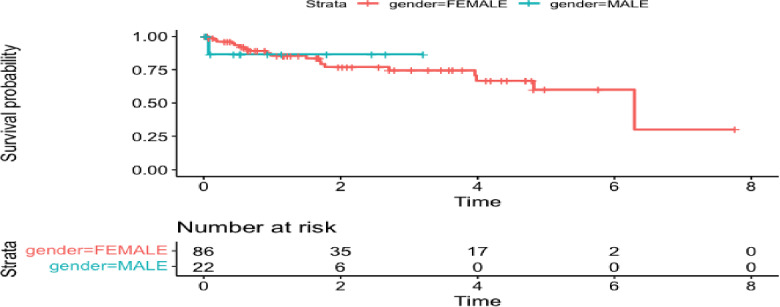
Plot of time to death by gender in local liver cancer data from Xu et al. [45]

The local dataset [45] we have consists of clinical measurements (estrogen recepter (ER) status, Barcelona status, local resection status) and followup times on 106 patients. Here, we use the TCGA database [28] to build risk prediction models for survival (time to death) in HCC patients. There are 106 and 377 observations in the local and TCGA datasets, respectively. We use age and gender to predict survival for the risk prediction models. The plot of survival by gender in the TCGA and local datasets are shown in Figures 4 and  5, respectively. We are going to analyze the associations of the following variables in the local dataset from Xu et al. [45]: BCLC stage, estrogen receptor status (positive/negative), and local resection (yes/no). We will treat BCLC stage as a continuous variable.

When running the proportional hazards model for prediction, all survival probabilities were estimated to be one so there was no difference between the weighted and unweighted analyses. We thus proceeded with using random forests for prediction, with weights being estimated using the cumulative hazard function. The results from the methods are presented in Table 3.

**Table 3:** Regression results for Liver Cancer Data. For these analyses, random survival forests have been used to compute weights using the TCGA HCC data from Liu et al. [28]

Variable	Proportional Hazards		Additive Hazards	
	Unweighted	Weighted	Unweighted	Weighted
BCLC	0.81 (0.31)	0.76 (0.31)	0.11 (0.06)	0.11 (0.05)
ER	1.08 (0.79)	0.91 (0.87)	0.09 (0.05)	0.08 (0.05)
LR	−1.12 (0.81)	−0.91 (0.90)	−0.09 (0.06)	−0.08 (0.06)

Based on the table, we see that irrespective of weights, using either proportional hazards or additive hazards leads to stronger evidence for the BCLC staging system as a predictor of survival relative to ER and LR status. Howver, we do note that using the weights from the external prediction model with age and gender leads to a slight reduction in the amount of evidence for the strength of BCLC staging.

## Discussion

In this article, we have presented a very simple strategy for modeling external information in the form of risk prediction models for analyzing datasets. The philosophy of the approach is to use the information as subject-specific weighting factors by which to adjust the local dataset for the analysis. Because the entire theory is based on model misspecification results [38, 43], there are minimal assumptions needed on the validity of the data generating processes, the model being fit to the data or to the compatibility across models. In addition, the model fitting procedures are relatively straightforward.

For the examples presented here, we have built risk prediction models using ‘off-the-shelf’ tools. The models have been optimized for classification. However, in practice, one should also be concerned about calibration. Well-calibrated models are those in which the predicted risk will match with the observed risk for individuals. The manner in which this typically is assessed is compared of the risk predictions from the model to some nonparametric (i.e., non model-based) estimate; the closer the predictions are, the better calibrated the model is. Calibration has been advocated in risk prediction by various authors [7, 8, 10]. As a matter of course, nonparametric estimates of risk models require binning of covariates or categorization of predicted values in order to deal with the inherent sparsity that exists with using continuous covariates.

## Appendix

### Results for Additive Hazards model with the Head and Neck Cancer dataset from MDACC

Table 4

**Table 4:** Additive hazards regression results for Head and Neck Cancer Data

Variable	Unweighted		PH-weighted		RSF-weighted	
	Estimate	SE	Estimate	SE	Estimate	SE
Radiomics Score	0.0014	0.001	0.0005	0.001	0.002	0.001
Age (55-65)	0.007	0.009	0.01	0.012	0.007	0.013
Age (65-75)	0.028	0.015	0.04	0.019	0.033	0.016
Age > 75	0.115	0.052	0.18	0.078	0.105	0.052
Male	$-$0.01	0.014	$-$0.02	0.019	0.001	0.02
T-stage 3	0.016	0.011	0.019	0.013	0.029	0.013
T-stage 4	0.045	0.016	0.088	0.024	0.053	0.017
Smoking status (never)	$-$0.008	0.013	$-$0.003	0.015	$-$0.015	0.016
Pack-years	0.0008	0.0003	0.0014	0.0004	0.001	0.0003
